# Resveratrol, Epigallocatechin Gallate and Curcumin for Cancer Therapy: Challenges from Their Pro-Apoptotic Properties

**DOI:** 10.3390/life13020261

**Published:** 2023-01-17

**Authors:** Adele Chimento, Maria D’Amico, Arianna De Luca, Francesca Luisa Conforti, Vincenzo Pezzi, Francesca De Amicis

**Affiliations:** 1 Department of Pharmacy, Health and Nutritional Sciences, University of Calabria, 87036 Rende, Italy; 2 Health Center, University of Calabria, 87036 Rende, Italy

**Keywords:** bioactive compounds, anti-cancer actions, chemoprevention, apoptotic cell death

## Abstract

Plant-derived bioactive compounds are gaining wide attention for their multiple health-promoting activities and in particular for their anti-cancer properties. Several studies have highlighted how they can prevent cancer initiation and progression, improve the effectiveness of chemotherapy, and, in some cases, limit some of the side effects of chemotherapy agents. In this paper, we provide an update of the literature on the anti-cancer effects of three extensively studied plant-derived compounds, namely resveratrol, epigallocatechin gallate, and curcumin, with a special focus on the anti-cancer molecular mechanisms inducing apoptosis in the major types of cancers globally.

## 1. Introduction

Chemotherapy represents one of the strategies available for cancer treatment [1]; it makes use of compounds capable of preventing proliferative signaling pathways, blocking mechanisms of immortality, preventing angiogenesis and metastasis, and directing cancer cells towards apoptosis [1,2]. Tumors exhibit a high degree of molecular and genetic heterogeneity which makes them extremely susceptible to cytotoxic drugs treatment [3]. 

Chemotherapy aims to kill cancer cells to prevent cancer progression. However, its efficacy is limited by the emergence of drug resistance mechanisms in tumor cells [4]. This may be due to increased drug efflux rates, drug inhibition and degradation mechanisms, drug target mutations, or death mechanism dysfunction [5,6]. In chemoresistance processes, all of these events can act independently or in synergy through the modulation of several signaling pathways [6]. Indeed, both intrinsic drug resistance existing before treatment and acquired drug resistance after therapy are responsible for most cancer recurrences, which are the leading causes of death from the disease [7]. Intrinsic drug resistance may be due to genetic mutation(s) of the genes involved in cancer cell growth and/or apoptosis. For example, the high expression of the HER2 gene renders cancer cells more resistant to cisplatin treatment in some tumors [8]. Furthermore, it may depend on the heterogeneity of tumors in which the existence of specific subpopulations in tumors such as CSCs with self-renewal and differentiation capacity participate in both tumor progression and resistance to chemotherapy drugs in different types of cancer [7,9]; the activation of intrinsic pathways used as a defense against environmental toxins (such as anti-cancer drugs) predisposes to chemoresistance. On the other hand, the acquired resistance after treatment, resulting from new proto-oncogenes activation, mutations or altered expression levels of specific drug targets, as well as changes in the tumor microenvironment, has been reported [7,9].

A promising strategy to fight cancer is chemoprevention which, uses natural bioactive compounds together with synthetic molecules, to block, inhibit, or delay cancer progression [10]. In recent decades, there has been a growing interest in the use of bioactive components from natural sources as potential anti-cancer agents to be added into combination therapy, along with the identification of molecular targets and signaling pathways activated or inhibited by them [11]. Studying the therapeutic effects of phytochemicals at the cellular and molecular behavior level provides the basis for their function and helps to potentiate the health benefits of these products.

Many existing anti-cancer drugs, such as the vinca alkaloids vincristine and vinblastine, taxanes (e.g., paclitaxel and docetaxel), podophyllotoxin derivatives (e.g., etoposide and teniposide), and camptothecins (e.g., topotecan) are molecules derived from plant origin which are used in conventional chemotherapy [12]. Furthermore, some natural bioactive compounds are considered as nutraceuticals, namely compounds isolated or purified from foods which provide benefits or protection against chronic diseases, including cancer [13]. Although nature offers an incredible diversity of compounds that could be proposed as candidates to counteract tumor growth, the chemical modifications of natural molecules can further enhance their efficacy and reduce negative effects [14]. A wide variety of bioactive compounds or natural product derivatives may be available in clinical practice. Results from several studies have suggested that the resistance against natural products is less frequent, due to their multi-targeting ability [15]. In fact, some of them not only influence the activity and expression of oncogenes, but are capable of activating the immune system response against tumors [15] and above all capable of inducing apoptosis [16]. Moreover, in addition to drug resistance, systemic toxicity is a serious problem inherent to chemotherapy. In vitro and in vivo studies have demonstrated that natural compounds have lower toxicity and also produce synergistic effects, with several common chemotherapeutic agents being able to simultaneously affect multiple cellular pathways [17]. Therefore, in cancer management, natural compounds can be used as both chemotherapeutic and chemo-preventive agents to prevent or treat cancer [17,18,19] and chemo-sensitizing agents to improve the efficacy of conventional chemotherapy [11,20].

**Figure 1:**
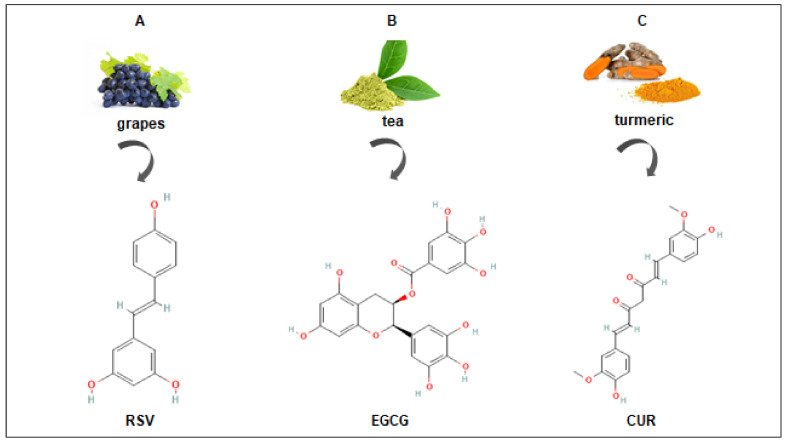
Chemical structures of resveratrol (RSV) (**A**), epigallocatechin gallate (EGCG) (**B**), and curcumin (CUR) (**C**), and relative natural sources. The chemical structures were taken from the PubChem Substance and Compound database. (https://pubchem.ncbi.nlm.nih.gov/) accessed on 20 December 2022.

The present review was designed to summarize reports regarding the potential use of natural bioactive products as chemo-preventive and chemo-therapeutic agents. These compounds are effective against several signaling pathways modulating cancer biology and mainly cell death apoptotic pathways [21]. Since apoptosis evasion is a fundamental feature of cancer cells, the ability of bioactive compounds capable of triggering this process can be utilized to treat or prevent cancer [22]. Due to the complexity of anti-cancer actions mediated by each bioactive compound, our analysis will focus on the pro-apoptotic actions of some specific polyphenols that are mostly under study, namely resveratrol (3,5,4′-trihydroxystilbene) (RSV), epigallocatechin gallate ((-)-cis-3,3′,4′,5,5′,7-hexahydroxy-flavane-3-gallate) (EGCG), and curcumin (1,7-bis[4-hydroxy-3-methoxyphenyl]-1,6-heptadiene-3,5,dione) (CUR). These bioactive compounds represent the most promising candidates for cancer chemoprevention or treatment (Figure 1).

## 2. Plant-Derived Bioactive Compounds

### 2.1. Resveratrol-Mediated Biological Actions

#### 2.1.1. Absorption and Metabolism of Resveratrol

Resveratrol (RSV) is a natural nonflavonoid polyphenol first characterized by its antioxidant cardiovascular beneficial effects, and subsequently by its anti-inflammatory and anti-cancer properties [23]. It is found in more than 70 plant species, although it is present in high concentrations in red grapes [24]. RSV consists of two aromatic rings that are connected through a methylene bridge (Figure 1). Two different isomers are defined: the cis- RSV form is unstable and the trans-isomer is characterized by greater biological activity, and stability and it is believed to be responsible for the anti-cancer and health benefits of RSV [25]. However, nowadays, further investigations are necessary to better define the potential clinical usefulness of RSV.

Unfortunately, RSV absorption in the intestine is very low due to its chemical structure. The extensive metabolism in the intestine and liver of the natural compound results in an oral bioavailability considerably less than 1% [26]. The majority of in vitro and in vivo studies have demonstrated that the poor bioavailability and low potency compromise the anti-cancer activity of RSV [27]. Thus, the high dose required to exert its biological effects is the remaining problem for its use in chemoprevention. To resolve these challenges, scientists have developed new carriers and covers for RSV [28,29] to overcome low bioavailability and absorption. Nano-encapsulated forms, liposomes, micelles, solid lipid nanoparticles, and polymeric nanoparticles are fascinating examples able to increase RSV bioavailability and absorption [30,31]. These options have improved its use for the preventive effect in several diseases, but also for the anti-tumor activity against various cancers.

#### 2.1.2. Anti-Cancer Pathways of Resveratrol

From this point of view, RSV is known to regulate multiple transduction pathways and enzyme activities that could induce different biological responses. For example, the suppression of the erroneous DNA double-strand breaks’ repair and genome stability maintenance have been described [32]. In an in vivo study, the beneficial effects of RSV on genome integrity were associated with the suppression of microsatellite instability-associated cancer and a subsequent extension of the lifespan of mice. These results strongly support the suppression of genomic instability as an anti-cancer mechanism through increased RSV consumption [32]. Moreover, RSV suppresses cancer cell survival, induces cell death, and is able to counteract metastasis and invasion in a number of cancer cell lines [33].

**Figure 2:**
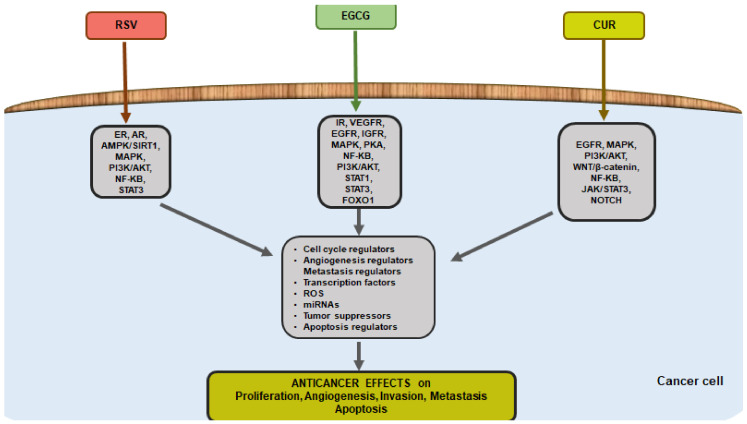
Schematic representation of the anti-cancer pathways stimulated by RSV, EGCG, and CUR in cancer cells. See the text for details.

Through numerous in vitro and in vivo studies (Figure 2), it has emerged that RSV exerts anti-tumor effects via pleiotropic mechanisms rather than a single mechanism of action [34]. More recently, its capability to act on multiple nodes, during tumor carcinogenesis strongly suggests the use of RSV as a combination agent with other therapies. RSV exerts synergistic action when combined with chemotherapeutic agents [35] or sensitizes [36] resistant tumor cells to cytotoxicity [37]. Indeed, a large number of receptors, kinases, and enzymes are described as RSV targets, mediating its biological effects.

Classically, RSV has been shown to target the steroid receptors signals in hormone-dependent cancer cells [38] thus suggesting that it might become part of future therapeutic protocols. RSV exerts anti-inflammatory actions mediated by its modulatory actions on AMPK/SIRT1; since SIRT1 may participate in mechanisms that delay age-associated diseases, this strongly suggests that RSV may have a similar function [39]. Moreover, RSV triggers the expression of a wide range of antioxidant enzymes, determining an overall decrease in oxidative stress [40].

Interestingly, further elegant studies provide evidence of novel RSV targets and anti-cancer mechanisms. In a study, the chronic administration of RSV induced a cell quiescent state with features of cell dormancy in ovarian cancer cells. The authors identified the signature of six miRNAs modulated in an opposite manner by IL-6 and RSV, potentially targeting ARH-I, a tumor-suppressor gene responsible for controlling the cancer cell dormant state. Additionally, the authors showed that ARH-I is a target of a novel onco-miRNA miR-1305, upregulated by IL-6 and downregulated by RSV [41].

More recently, a total of 330 genes with significantly different expressions were identified through large-scale transcriptome sequencing. These data proved that a large number of genes related to the cell cycle and apoptosis were differentially expressed after RSV treatment [42,43].

### 2.2. Epigallocatechin Gallate-Mediated Biological Actions

#### 2.2.1. Absorption and Metabolism of Epigallocatechin Gallate

EGCG is a common phytochemical rich in biologically active components and it is the most studied molecule belonging to the flavonol class. It is a catechin conjugated with gallic acid (Figure 1) and is considered to be one of the most active molecules known for its anti-oxidant properties. EGCG is particularly abundant in green tea (Camellia sinensis), the second most consumed beverage in the world [44]. Numerous observational and intervention studies have linked green tea consumption to beneficial effects counteracting many human diseases, including obesity, metabolic syndrome, neurodegenerative inflammatory diseases, and cancer [45]. In the past 25 years, the cancer preventive and other anti-cancer activities of tea preparations have been demonstrated in a variety of animal models [46], including those for cancers of the oral cavity, esophagus, stomach, small intestine, colon, liver, pancreas, lung, bladder, skin, prostate, and mammary glands [47].

Most of the in vivo studies were conducted with green tea or tea catechin preparations, and some were conducted with pure EGCG, administered through drinking water or diet [48]. EGCG is first taken up in the intestine where the intestinal microbiota plays an essential role in the metabolism of EGCG. The gut microbiota is a key factor in maintaining the intestinal barrier function, which is crucial for nutrient absorption, energy metabolism, and immune response regulation [49]. After the ingestion of green tea or EGCG, only a very small amount of EGCG appears in the systemic circulation, while most reaches the large intestine, where it is degraded by the intestinal microbiota [50]. Several in vitro and in vivo studies have reported that gut microbiota can deconjugate and degrade EGCG; microbial conversion begins with rapid degalloylation of the D-ring by microbial esterases, resulting in gallic acid (GA) and epigallocatechin (CGE). Subsequently, these two metabolites are subjected to further degradation [51]. In animal models, the radioactivity of isotope-labelled EGCGs increases only eight hours after oral administration, indicating that the intestinal microbiota is responsible for significant conversions of EGCGs prior to uptake [52].

#### 2.2.2. Anti-Cancer Pathways of Epigallocatechin Gallate

By attaching to lipids and proteins found in membranes, EGCG is able to perform a variety of biological functions and target different pathways (Figure 2) [53]. EGCG regulates the activities of cell surface growth factor receptors, such as IR, VEGFR, EGFR, and IGFR [54]. Several intracellular messengers including Ca^2+^ and cGMP are increased by EGCG. Even though this effect is not seen in other cell types, EGCG stimulation increases cAMP in endothelial cells and platelets. The increased cAMP induces PKA which has a variety of biological effects [55] in different cell models. By blocking a number of transcription factors, including Sp1, NF-kB, AP-1, STAT1, STAT3, and FOXO1, EGCG modulates gene expression [56].

EGCG’s anti-cancer activity is continuously investigated [57]. EGCG has been shown to have chemo-preventive effects through the inhibition of the initiation, promotion, and development of carcinogenesis [58]. Additionally, through modifying several cell signaling pathways, such as those controlling proliferation, apoptosis, and angiogenesis, this catechin has demonstrated its significance as a promising compound for the treatment of different types of cancer [58]. EGCG has been shown to have an additive or synergistic impact with chemo-preventive drugs because it lessens toxicities and strengthens anti-cancerous benefits [59]. EGCG has a number of advantages, but there are also a growing number of obstacles to overcome. For instance, oral administration of EGCG results in minimal bioactivity [60].

Some of the anti-cancer effects of catechins may be related to the production of oxidative stress and the stimulation of apoptosis in tumor cells caused by these pro-oxidant actions. These pro-oxidant actions may also activate endogenous antioxidant defense mechanisms in healthy tissues, which provide defense against carcinogenic damage [57].

### 2.3. Curcumin-Mediated Biological Actions

#### 2.3.1. Absorption and Metabolism of Curcumin

Curcumin (CUR) is the most active agent of the polyphenolic curcuminoids derived from turmeric root. Turmeric includes one of the largest genera of the Zingiberaceae family (about 133 species) [61,62], which is widely distributed in tropical regions from India to Southern China and Northern Australia [61]. The most interesting species is *Curcuma longa* L., particularly cultivated in India; a powder with a characteristic yellow-orange color is obtained from the underground root (rhizome) of this plant, after drying and grinding. This extract contains various active ingredients including volatile oils (i.e.,α-zingiberene, curlone, and α-turmerone) [63] and mainly (95% of standardized extract) three curcuminoids such as CU (or CUR I) (~ 77%), demethoxy CUR (17–18%) (or CUR II), and bis-demethoxy CUR (3–5%) (or CUR III) [64]. CUR is a symmetric molecule that possess three chemical parts in its structure: an aromatic o-methoxy phenolic group, α, β-unsaturated β-diketo moiety, and a seven-carbon linker (Figure 1).

After oral intake, CUR is poorly absorbed and rapidly metabolized [65]. The liver represents the primary site of CUR metabolism, although it can be transformed in the intestine [66]. CUR undergoes extensive phase I and II biotransformation reactions [67]; phase I metabolism involves the reduction of double bonds leading to the formation of dihydro, tetrahydro-, hexahydro-, and octahydro-CUR, while phase II metabolism conjugates glucuronide or sulfate with CUR and its hydrogenated metabolites [67]. CUR’s water solubility can be increased by glucuronidation and sulfation, even if these reactions accelerate CUR removal via urine [68]. Indeed, the poor absorption, high rate of metabolism, inactivity of metabolic products, low plasma levels, tissue distribution, and rapid elimination and clearance from the body contribute to CUR’s poor bioavailability and all together, they limit its clinical application [69]. In this regard, several approaches to improve its pharmacokinetic profile and bioavailability have been performed; these include the discovery of synthetic derivatives/analogues and various drug delivery systems [62,70,71,72,73,74,75].

#### 2.3.2. Anti-Cancer Pathways of Curcumin

Despite its unfavorable pharmacokinetic characteristics, growing evidence has shown that CUR has numerous pharmacological properties, including antimicrobial [76], antiviral [77], antifungal [78], antioxidant [79], anti-inflammatory [80], and also anti-tumoral properties [81]. In particular, CUR represents a promising phytochemical in the anti-cancer field to be used alone or combined with other drugs through different pathways (Figure 2). In vitro and in vivo studies have evidenced that CUR may inhibit breast, lung, gastrointestinal tract, liver, prostate, kidney, pancreatic, leukemia, osteosarcoma, melanoma, and brain cancer growth [82,83]. In addition, CUR is able to potentiate the anti-cancer effects of both radiotherapy and chemotherapeutic agents [84] showing low toxicity towards normal cells. Co-treatment of CUR with chemotherapy drugs such as docetaxel, metformin, 5-fluorouracil, doxorubicin, cisplatin, and celecoxib improved their anti-tumor actions through a synergistic effect in several tumors such as prostate, hepatocellular, gastric, lymphoma Hodgkin’s, bladder, and colorectal tumors [85]. Extensive data suggests that this polyphenol can effectively prevent and treat cancer at the initiation, promotion, and progression levels [86]. This propriety depends on its ability to target critical processes primarily involved in cancer progression. CUR was able to modulate several cellular pathways affecting proliferation and/or angiogenesis, invasion, migration, metastasis, and apoptotic processes [62,81]. Change in the expression of tumor suppressor, pro-apoptotic, and anti-apoptotic genes by CUR represents one of the key molecular mechanisms underlying its anti-cancer action [87]. Studies have demonstrated that it can affect p53, JAK/STAT, EGFR, PI3K/Akt, MAPK, NOTCH, Wnt/β-catenin, and NF-ĸB apoptosis-related signaling pathways. Recent data has indicated that it also modulates the expression of ncRNAs (including miRNAs and lncRNAs) and the expression of specific proteins thus inhibiting tumor proliferation, promoting apoptosis, and improving sensitivity to chemotherapy drugs [87,88].

## 3. Plant-Derived Bioactive-Compound-Mediated Pro-Apoptotic Effects

Apoptosis has been reported to be hindered in many human cancers, showing that the disruption of apoptotic processes plays a crucial role in cancer development. Apoptosis-programmed cell death is a physiological process controlled by genetics that helps the body get rid of damaged or diseased cells. Apoptosis evasion is retained nowadays as one of the hallmarks of cancer. There are many ways by which cancer cells evade apoptosis: caspase function can be inhibited or the switch for apoptosis can be disabled. The upregulation of anti-apoptotic Bcl-2 proteins and the loss of Bax and/or Bak are the predominant methods of evasion and represent several mechanisms of resistance to the apoptotic stimuli of some anti-cancer drugs [89].

The mitochondria-apoptosome-mediated intrinsic pathway and the death receptor-induced extrinsic pathway are the two main mechanisms that are involved in the start of apoptosis [90]. The intrinsic process relies on mitochondria and can destroy injured cells by detecting DNA damage and oxidative stress. Intrinsic pathways rely on the tumor-suppressor protein p53, which is essential in this route. As a result, p53 is classically thought to be a potential target for cancer treatment. In order to activate the extrinsic apoptotic pathway, death ligands bind to the death receptors. TNF was the first cell death ligand investigated for cancer treatment [91] and many others have been described subsequently. Recently, it was discovered that the Fas/APO-1 (CD95) receptor is an important cellular component responsible for the induction of apoptosis in leukemic cells and an important therapeutic target, because antibodies directed against this receptor were able to cause apoptosis in a variety of cancer cells. Numerous studies illustrate the mechanisms and targets of RSV, EGCG, and CUR anti-apoptotic actions and they summarized in Table 1.

### 3.1. Resveratrol as an Enhancer of Apoptosis in Cancer Cells

#### 3.1.1. Pro-Apoptotic Pathways of Resveratrol

RSV induces the apoptotic death of tumor cells through a variety of signal transduction mechanisms by regulating the amounts of Fas and FasL. In HL-60 promyeloblast cells, RSV increases FasL expression, and RSV-induced apoptosis is dependent on Fas signaling [111]. Similar results have been seen in breast and colon cancer cells [112]. However, cytotoxic chemicals that kill cells without targeting the Fas protein have also been described [113]. The RSV-induced death of THP-1 monocytes cells is independent of the Fas/FasL signaling system, and it at least partially inhibits the proliferation of THP-1 cells. RSV acts differently on monocytes and macrophages since it enhances the inflammatory response in THP-1 monocytes, whereas it exerts anti-inflammatory effects in differentiated-THP-1. This study shows that RSV exerted a concentration-dependent differential effect on cell proliferation and apoptosis depending on cell types, with monocytes being more sensitive toward RSV than macrophages [114]. Thus, the potential use of RSV could be now considered for the treatment of acute monocytic leukemia [115]. In a T-cell-derived T-ALL lymphocytic leukemia cell line, which lacked functioning p53 and p16, RSV caused apoptosis and an arrest in the S-phase, in a way that was Fas-independent as evidenced by the lack of an apoptotic change in the presence of Fas or FasL antagonist antibodies [116].

Classical mechanisms of action were described in human multiple myeloma cells which had undergone apoptosis and were prevented from proliferating by RSV, through downregulation of specific anti-apoptotic and pro-proliferation gene products such as survivin, cIAP-2, cyclin D1, XIAP, Bcl-xL, Bfl-1/A1, and TRAF2 [117]. RSV has been demonstrated to cause cell death in some cancer cells by altering Bcl-2 family proteins. By blocking the anti-apoptotic proteins Bcl-xL, Bcl-2, and Mcl-1, activation of proapoptotic BH3-only members of the Bcl-2 family (Bak and Bax) impairs mitochondrial outer membrane permeability [118].

#### 3.1.2. Targets of Resveratrol’s Pro-Apoptotic Action

##### P53

**Figure 3:**
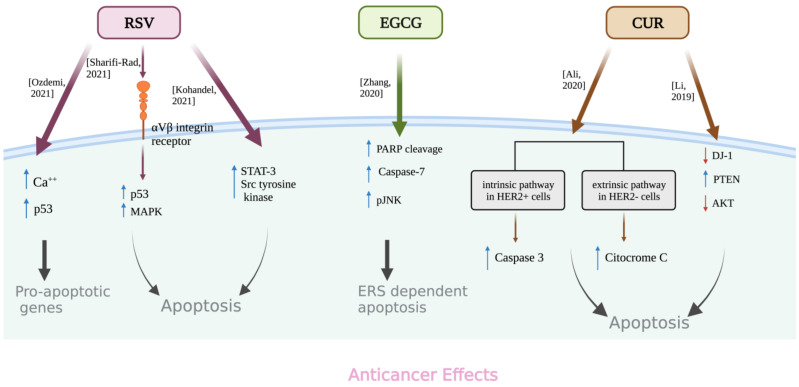
Schematic representation of the apoptotic mechanisms exerted by RSV, EGCG, and CUR in breast cancer cells. See the text for details. ↑ (increase); ↓ (decrease); Özdemi,2021 [121]; Sharifi-Rad, 2021 [122]; Kohandel, 2021 [127]; Zhang, 2020 [129]; Ali, 2020 [130]; Li, 2019 [131].

The activation of p53 may in an intriguing way be strictly related to apoptotic mechanisms. One of the most important aspects of p53’s tumor-suppressor role is its capacity to induce growth arrest or triggering apoptosis in response to a variety of cellular stressors, including DNA damage, oncogenic activation, hypoxia, etc. [119,120]. Cancer cells are able to bypass typical stress reactions when p53-dependent processes are defective. RSV enhanced the cytoplasmic content of calcium in human breast cancer MDA-MB-231 cells, which activated p53 and induced the transcription of many pro-apoptotic genes [121] (Figure 3).

##### MAPK

By transmitting extracellular signals to intracellular reactions, MAPKs control a variety of cellular activities. Although constitutively active MAPKs are required to sustain the malignant state, short-term MAPKs activation may cause the cells to undergo apoptosis. RSV triggered apoptosis in breast cancer cells through binding to integrin αVβ3, activating the MAPK- and p53-dependent pathways [122] (Figure 3). RSV simultaneously stimulates MAPKs in human neuroblastoma SH-SY5Y cells at low concentrations, while at higher concentrations, it can inhibit MAPKs. However, in prostate, breast, glial, head and neck, and ovarian cancer cells, RSV stimulates the same signal [112].

RSV has also been linked to the activation of other kinase members, including JNK and MAPKp38 [92]. Interestingly, it has been demonstrated that the anti-tumor actions of RSV and the induction of apoptosis necessitate p53 activation, which is MAPK-induced. JNK and the p38 MAPK apoptotic signal mediate the RSV regulation of IGF-II, a potent mitogen and apoptosis inhibitor. IGF-II regulation is complicated, involving tumor suppressor inhibition, oncogene promotion, and hormonal modulation by estrogens in breast cancer. IGF-II was shown to prevent RSV’s apoptotic action in breast cancer cells [123]. The mechanism can be dependent on the formation of ROS and lipid oxidation, followed by the activation of JNK and the p38 MAPK apoptotic signal [124].

##### PI3K/Akt

RSV inhibits the PI3K/Akt/mTOR signaling pathway through regulation of MAPK. The inhibitors of these signaling proteins further increase the activation of caspase-3 and cell death that is brought on by RSV. According to these studies, RSV inhibits Akt activation and expression and causes apoptosis in human glioma cells [93]. In ovarian, breast, uterine, prostate, and multiple myeloma cells, RSV also inhibited Akt phosphorylation, causing apoptosis [125]. This led to the stimulation of Bim, TRAIL, p27/KIP1, DR4 and DR5, suppression of cyclin D1, and a decrease in the activation of FOXO in prostate cancer cells [94].

##### NF-kB/STAT3

In addition to being pro-apoptotic and anti-proliferative, RSV also inhibits NF-kB and STAT3 which encourage the survival, invasion, angiogenesis, and metastasis of tumor cells, and which are key players in the development of inflammation-related carcinogenesis. STAT3 and NF-κB once activated travel to the nucleus to trigger transcription through several mechanisms. Intriguingly, medulloblastoma cells treated with RSV underwent apoptosis when NF-κB nuclear translocation was inhibited [95]. NF-κB-regulated gene products also including those linked to inflammation were suppressed by RSV [126].

The activation of the STAT3 together the biochemical pathway modulated by Src tyrosine kinase are the target of RSV in both triple-negative and ER-positive breast cancer cells inducing apoptosis [127] (Figure 3).

##### miRNAs

RSV combined with conventional chemotherapy drugs enhances chemotherapy efficacy, in particular reducing both chemotherapy toxicity in normal tissues and side effects. For instance, RSV efficiently increased the sensitivity of MCF-7 cells to adriamycin in a dose-dependent way, resulting in a lower IC50 value and an increase in the number of apoptotic cells. The authors demonstrated that RSV specifically targeted their main target proteins. The modification of the key suppressor, miR-122-5p, caused RSV-induced chemosensitivity and apoptosis. Interestingly, miR-122-5p inhibitors showed that miR-122-5p had a significant influence on the regulation of critical anti-apoptotic proteins Bcl-2 and Cdks in drug-resistant breast cancer cells, in response to RSV [128].

### 3.2. Epigallocatechin Gallate as an Enhancer of Apoptosis in Cancer Cells

#### 3.2.1. Pro-Apoptotic Pathways of Epigallocatechin Gallate

EGCG promotes apoptosis in different cancer cells in a concentration-related manner even though the effects on normal cells were less prominent than the effects exerted on sensitive neoplastic cells [58]. Specifically, EGCG induced apoptosis, decreased mitochondrial membrane potential, and promoted the G0/G1 phase cell cycle arrest of liver carcinoma cells but not that of non-cancerous liver cells. EGCG-induced apoptosis of cancer cells has been linked with a substantial decrease in Bcl-2 expression [96], caspase-8 activation, and proteolytic cleavage of Bid in glioblastoma cells. However, EGCG did not promote apoptosis in astrocytes [97]. EGCG induced Bcl2-mediated apoptosis by targeting EZH2 which plays a critical role in tumorigenesis. Specifically, this study suggests that EZH2 could function as an inhibitor of the mitochondrial apoptosis pathway during EGCG-induced apoptosis [132]. Green tea and EGCG have been reported to present pro-apoptotic effects in myeloid leukemia cell lines as well as in in vivo models [132]. Green tea (whole extract) decreased leukocytosis and promoted a reduction of immature cells in the bone marrow and spleen of acute promyelocytic leukemia xenografts by activation of caspase-3, -8, and -9 [99]. The authors concluded that EGCG reduced leukaemia immature cells and promyelocytes in the bone marrow while increasing mature myeloid cells, through apoptosis increase and cell differentiation.

#### 3.2.2. Targets of Epigallocatechin Gallate’s Pro-Apoptotic Action

##### PI3K/Akt

EGCG promotes apoptosis targeting the PI3K/Akt pathway in bladder cancer cells [98]. Two different cell lines were treated using several concentrations of EGCG. The data demonstrated that EGCG suppresses cell proliferation and increases apoptosis in bladder cancer cells in a dose-dependent manner. The authors concluded that low doses of EGCG lead to inactivation of the PI3K/AKT/mTOR pathway and induced autophagy-related apoptosis [100]. Further evidence demonstrates that EGCG caused significant apoptosis and inhibited the migration and invasion of colon cancer cells. The EGCG stimulus had low toxicity against normal colon epithelial cells although it exerted an antiproliferative effect against colon cancer cells in a dose-dependent manner. The toxic effect on colon cancer cells was linked to the downregulation of the major trafficking networks such as the Shh signaling and PI3K/Akt pathways. Very interestingly, the results obtained in in vivo models confirmed these findings. Indeed, EGCG inhibited the activation of the Shh and PI3K/AKT pathways in tumor tissue and reduced tumor volume and weight without affecting the body weight of nude mice [101].

##### ROS

Although a huge number of molecular targets for EGCG’s biological effects have been reported, the mechanisms of its anti-cancer activities need to be clarified. Classically, EGCG can induce the production of ROS in cancer cells and induce apoptosis, but paradoxically it may exert an antioxidant action, decreasing ROS and inhibiting cancer development [133]. EGCG modulates the activities of receptors, signaling molecules, and enzymes thus affecting apoptosis. Recent evidence has reported the effect of EGCG on the activity of PIN1 [134], an enzyme commonly over-activated in most human cancers, that binds to and catalyzes the conversion of proline-directed serine/threonine phosphorylation, thus altering the balance of oncogenes and tumor suppressors promoting oncogenesis [135]. EGCG decreases the expression of PIN1 and substrate oncoproteins for PIN1, together with the ROS amount. The authors showed a reduction in the spleen weights of mice models and the apoptosis of spleen cells, supported by increasing expression of Bad and Bax, matched to the Bcl-2 and c-Myc decrease [134].

##### ERS

Apoptosis may be induced by ERS as a cellular stress response which is due to several factors. EGCG prevents tumor cell growth via ERS-induced apoptosis in colorectal cancer cells by upregulating BiP, PERK, phosphorylation eIF2a, ATF4, and IRE1a and by increased caspase-3/7 activity [102]. Glucosidase II, which contributes to quality control and glycoprotein processing in the endoplasmic reticulum of rat liver microsomes, is inhibited by EGCG, thus interfering with protein processing. A progressive elevation of apoptotic activity was also observed as well as the cleavage of procaspase-12 and the increasing phosphorylation of eIF2alpha while the induction of endoplasmic reticulum chaperones was not observed [136]. GRP78 is a molecular chaperone of the endoplasmic reticulum that aids proper folding of nascent polypeptides. In mesothelioma cells, EGCG treatment improved GRP78 expression in the endoplasmic reticulum, and increased the activity of caspase-3 and caspase-8. In this way, EGCG through GRP78 accumulation converted the unfolded protein response into pro-apoptotic ERS [103]. This argues for a possible therapeutic use of EGCG in the treatment of mesothelioma to be added to the conventional portfolio of drugs.

EGCG also promotes the sensitivity of cancer cells to therapeutic drugs. GRP78 silencing, but also EGCG treatment, improved apoptosis induced by celecoxib, a selective cyclooxygenase-2 inhibitor that has been reported to elicit anti-proliferative response in various tumors, thereby activating caspase-4, in urothelial carcinoma cells [137]. In breast cancer models EGCG enhanced the ERS-dependent apoptosis response of vinblastine and Taxol through the activation of PARP cleavage, caspase-7, and JNK phosphorylation [129] (Figure 3). However, severe antagonism during combination drug treatments has been described. For instance, EGCG, but also green tea extract, effectively blocked proteasome inhibition by bortezomib in clinical use for multiple myeloma and thereby prevented ERS and subsequent tumor cell death in vitro and in vivo. [138].

### 3.3. Curcumin as an Enhancer of Apoptosis in Cancer Cells

#### 3.3.1. Pro-Apoptotic Pathways of Curcumin

Several studies have confirmed a CUR-mediated extrinsic apoptotic pathway. The Fas ligand and Fas increase at both mRNA and protein levels along with caspase-3 and PARP-1 activation after CUR have been observed in human hepatocellular carcinoma Huh7 cells [139]. Moreover, Fas/caspase-8 pathway activation was demonstrated in melanoma [140] and human gastric (KATO III) and colon (HCT-116) cancer cells [141] where CUR also caused PARP-1 cleavage and caspase-3 activation. A CUR-mediated extrinsic apoptotic pathway by upregulating the DR5 protein was observed in HCT-116 and HT-29 colon cancer cells [104]. Recently, Ali and coworkers elucidated the mechanisms of CUR-induced apoptosis in human EGFR-positive (BT474) and -negative (MCF-7) breast cancer cell lines [130] (Figure 3). The authors demonstrated that while CUR activated the mitochondria-independent extrinsic pathway in BT474 cells without cytochrome c release into the cytoplasm, in MCF-7 cells it activated a mitochondria-dependent pathway with cytochrome c release. In addition, it significantly upregulated the caspase-8 activity but not caspase-9 in both BT474 and MCF-7 cells, and that of caspase-3 only in BT474 cells, since MCF-7 cells do not express it [130]. Moreover, in the colon carcinoma cell line SW480, CUR-mediated apoptosis occurred by caspase-8 activation via FADD, and, ultimately, by activation of effector caspase-3 [142].

However, from the available in vitro data, it appears that CUR induces apoptosis in cancer cells mainly through mitochondrial pathway activation. In acute lymphoblastic leukemia cell lines, CUR suppresses cell viability through an intrinsic apoptotic mechanism [143]. In particular, it caused AKT and IAP’s downregulation, increased the Bax/Bcl-2 ratio and cytochrome c release from the mitochondria to the cytoplasm, and activated caspase 3 and PARP-1 cleavage and ROS production [143]. The CUR-mediated intrinsic apoptotic pathway was also demonstrated in a neuroblastoma SK-N-SH cell model [144].

#### 3.3.2. Targets of Curcumin’s Pro-Apoptotic Action

##### P53

The role of p53 in CUR-mediated ROS generation and cell death was evaluated in mutated p53 HT-29 and wild-type p53 HCT-116 colon adenocarcinoma cell lines. In both of these cell lines, CUR induced ROS production and inhibited MMP; moreover, p53 and cleaved caspase 3 protein expression increase was found in HCT-116 cells after CUR treatment [145]. CUR’s anti-tumor effects were related to inhibition of Notch3 expression and induction of p53 in mouse myeloma cells. This event was associated with Notch3-responsive Hes family BHLH transcription factor 1 and a Hes-related family transcription factor with YRPW motif 1 genes expression increase; in these cells, CUR treatment also significantly downregulated Bcl-2 and upregulated Bax at both mRNA and protein levels [105].

##### Wnt/β-Catenin

CUR exerts anti-proliferative and pro-apoptotic effects in SNU-1, SNU-5, and AGS gastric cancer cells and in the AGS gastric xenograft model via Wnt/β-catenin signaling pathway inhibition and subsequently Wnt target genes expression reduction [106]. Similarly, it induced apoptosis in BEL-7402 and QGY-7703 human hepatocellular carcinoma cell lines and blocked Wnt signaling by decreasing β-catenin target genes expression [107].

##### JAK/STAT3 and NFkB

A recent study demonstrated CUR-mediated apoptosis through targeting of the JAK/STAT3 signaling pathway in BCPAP and TPC-1 papillary thyroid cancer cell lines and derived thyroid cancer stem-like cells. In particular, it inhibited JAK/STAT3 signaling pathway constitutive activation and increased ROS production leading to apoptosis. Indeed, the observation that NAC, a ROS scavenger, reversed CUR-mediated caspase-3 activation, Bcl2 and Bcl-xL decrease, and Bax increase confirmed the role of oxidative stress in CUR-triggered apoptosis [108].

NFkB is another target of CUR which together with tumor-necrosis-factor-alpha-related apoptosis-inducing ligand (TRAIL) induced apoptosis via inhibition of NFkB activity and enhancement of caspase-3 activity in chronic myeloid cells [146].

##### PI3K/AKT and MAPK

EGFR, PI3K/AKT, and MAPK pathways’ hyperactivation is associated with drug resistance and cancer progression [147,148,149,150]. An interesting recent study suggests that CUR might be an encouraging therapeutic candidate for vemurafenib-resistant melanoma [109]. CUR suppressed cell proliferation and triggered apoptosis by downregulating the protein phosphorylation levels of EGFR, AKT, and ERK; furthermore, the combined use of CUR and gefitinib, an EGFR-targeting inhibitor, synergistically potentiated the inhibitory effect on cell viability, suggesting a role for the EGFR signaling pathway in CUR-induced apoptosis in vemurafenib-resistant melanoma cells [109]. Moreover, in these cells, CUR triggered apoptosis via ROS production increase, MMP disruption, and intrinsic signaling caspase-9/-3-dependent pathways activation [109].

##### PTEN

Wang and coworkers demonstrated that CUR inhibited proliferation by decreasing the p-AKT/p-mTOR pathway and induced apoptosis by increasing the tumor suppressors PTEN in U251 and U87 glioblastoma cell lines [151]. The upregulation of DJ-1, a negative regulator of PTEN, is associated with various tumor occurrences [152]. In A549 lung cancer cells, CUR inhibited cell proliferation and promoted apoptosis by downregulating DJ-1 [110]. The same effects were observed in MDA-MB-231 and MCF-7 breast cancer cells where CUR inhibited breast cancer cell proliferation and facilitated cell apoptosis via downregulation of DJ-1 expression and consequently through PTEN/PI3K/AKT pathway modulation [131] (Figure 3). The important role of DJ-1 in CUR-mediated apoptosis was demonstrated in SW480 and SW620 colorectal cancer cells [153].

##### miRNAs

miR-21 [154,155] is frequently upregulated and functions as an oncogene in several cancers [156]. miR-21 inhibition can mediate CUR’s anti-cancer effects including apoptosis through PTEN/PI3K/Akt, PDCD4, and NF-κB [157]. The results obtained from studies on an MGC-803 gastric cancer cell line suggested that CUR induced apoptosis by inhibiting the miR-21/PTEN/Akt molecular pathway [158]. CUR-dependent apoptosis via miRNA modulation was demonstrated as in RT4 schwannoma cells [159]. Further evidence obtained by microarray analysis from CUR-treated cells revealed that miRNA 344a-3p was significantly upregulated; interestingly, when the cells were transfected with a miRNA 344a-3p mimic, Bcl2 mRNA expression decreased and that of Bax increased. Moreover, the miRNA344a-3p mimic combined with CUR treatment enhanced cleaved caspase-9 and -3 activation [159]. Furthermore, the upregulation of miR-99a was observed in CUR SO-Rb50 and Y79 retinoblastoma-treated cells [160]. This molecular event was related to JAK/STAT pathway inhibition on which CUR’s anti-tumor activities and apoptotic events depend [160].

## 4. Limitations of Bioactive Compounds’ Use in Chemoprevention and Clinical Trials

The proposed use of natural products for chemoprevention imposes several challenges that must be overcome. Among these, the bioavailability of the natural compound, its half-life within the organism, and the elimination pathways represent the ones that mostly influence the therapeutic application [161]. In particular, poor bioavailability is the main limiting factor found in chemoprevention studies. To date, many efforts have been made to improve the pharmacokinetic characteristics of bioactive compounds, through new delivery and release systems and new analogues synthesis, which manage to reach their preferred action site in an almost unchanged way [161]. Recently, the application of nano-pharmaceutical formulations has been discussed, such as solid lipid nanoparticles, nano-emulsions, nano-crystals, nano-polymersomes, liposomes, ethosomes, phytosomes, and invasomes to overcome poor bioavailability and metabolic instability for bioactive compounds. The efficacy of different therapeutic nanodrug delivery systems depends on their properties, the characteristics of the loaded compounds/agents, and their expected therapeutic applications. However, there are still significant obstacles to apply this technology; these include the absence of trials to manage the interactions of nanomaterials with biological systems and the targeted efficiency of nanoparticulate drug delivery as well as regulatory aspects for toxicity profiles and biocompatibility [162].

Following the discovery of less toxic and highly active natural products as anti-cancer agents, the possibility of extending chemoprevention with such substances to a larger population may be offered. However, educating a large population in lifestyle choices that favor foods rich in natural bioactive compounds is not always an easy goal to achieve [163]. In addition, since most bioactive compounds are naturally present in the diet, a reduction in side effects is likely to occur. However, since the doses given for chemoprevention purposes and those in the diet are likely to be very different, as yet unknown side effects may happen. In this regard, it is necessary to implement clinical trials to test the doses that are therapeutically effective and to evaluate any adverse effects.

Several clinical studies have confirmed the anti-cancer actions of RSV, EGCG, and CUR, suggesting the potential clinical application for them (Table 2).

The efficacy, safety, and pharmacokinetics of RSV have been documented in clinical trials; however, there are only a total of seven clinical trial publications on RSV compared with more than 3000 publications on preclinical studies. These studies showed that it potentially improves the therapeutic outcome in patients suffering from colorectal cancer, breast cancer, and multiple myeloma [164] through several targets; its efficacy depends on the type and stage of cancer, dosage levels, and treatment periods. RSV is more effective in certain types of cancer than in others; it seems to epigenetically reduce the expression of certain breast-cancer-related genes, but it caused severe adverse events specifically in multiple myeloma patients. Therefore, the vast amount of preclinical data in support of RSV’s use as a chemo-preventive or chemotherapeutic agent warrants further clinical studies [165].

In addition, several clinical studies on human samples/subjects have proven that EGCG functions in cancer treatment and prevention [166]. About 40 clinical trials have been conducted on patients affected by different types of cancer and about twenty of these were completed (https://clinicaltrials.gov/ (accessed on 25 November 2022)). Some results confirmed that EGCG could prevent the cell growth of certain malignancies such as prostatic ones [167]. A randomized clinical trial of brewed green and black tea in men with prostate cancer prior to prostatectomy showed that daily consumption of brewed green tea increased tea polyphenols uptake in the prostate gland and decreased nuclear NFkB and systemic oxidant activity [168]. A phase I clinical trial with EGCG and standard chemotherapy (etoposide and cisplatin) and thoracic radiotherapy in unresectable stage III non-small cell lung cancer showed that oral administration of EGCG is safe and effective and suggested that it could be act as radioprotector without affecting the efficacy of radiation [169]. Moreover, a recent randomized clinical trial confirmed that EGCG solution use significantly reduced the incidence and severity of radiation-induced dermatitis in patients undergoing adjuvant radiotherapy for breast cancer [170]. However, other human clinical trials are still required to establish the efficacy of EGCG in the treatment of cancer.

A recent study analyzed clinical trials conducted from 2010–2020 related to the effect of CUR and its metabolites on different types of cancers, such as chronic myeloid leukemia, multiple myeloma, prostate, colorectal, and pancreatic cancer including complications related to cancer therapy [171]. From this study it emerged that sixteen out of twenty-one clinical trials were associated with the efficacy of CUR or turmeric on various types of cancer while five confirmed their efficacy in relieving the side effects of chemotherapy and radiotherapy [171].

**Table 2:** Representative clinical trials with RSV, EGCG, and CUR investigating their effects in cancer treatment. ↓ (decrease); ↑ (increase); GT (green tea); BT (black tea).

Phytochemical Compound/Formulation	Cancer Type	Dosage and Treatment (Time)	Patients (Number)	Outcomes	Ref.
RSV	Colon	20 and 80 mg/day; 14 days	8	↑ Myc, axinII, and cyclin D1; ↓CD133 and LGR5 in normal colonic mucosa	[164]
RSV	Breast	10–50 mg/day; 12 weeks	39	↓ RASSF-1α methylation	[164]
Micronized RSV	Colorectal	5 g/day; 14 days	9	Improved RSV bioavailability; ↑Cleaved caspase-3 in malignant hepatic tissue	[164]
EGCG (from GT and BT polyphenols)	Prostate	562 mg of EGCG from 1010 mg of GT polyphenols; 28 mg of EGCG from 80 mg of tea polyphenols; 2–8 weeks	113	↑ Tea polyphenols uptake in the prostate gland; ↓ NFkB and systemic oxidant activity	[168]
EGCG+chemotherapy and thoracic radiotherapy	Non-small cell lung	40–440 μmol/L; 15–58 days	24	Low EGCG toxicity, esophagitis regression, ↓pain score	[169]
EGCG+ radiotherapy	Breast	660 μmol/L; 2 weeks	180	↓ Radiation-induced dermatitis	[170]
CUR	Prostate	3 g/day; 7–8 weeks	40	↓ PSA levels	[171]
CUR-based gel	Breast	curcumin-based gel; 4–6 h/week	191	Intensity decrease of radiation-induced dermatitis	[171]
CUR microgranular	Head and neck	4 g twice daily; 21–28 days	33	Good CUR tolerability; ↑ CUR bioavailability	[171]

## 5. Conclusions

The current review has provided an overview of the latest mechanistic insights into the anti-cancer apoptotic action of RSV, EGCG and CUR. These dietary bioactive compounds exhibit pleiotropic, multi-target apoptotic activities and, for their anti-cancer properties, are emerging as promising molecules to be added in the current therapeutic portfolio of different cancer types. Testing these compounds in combination with chemo- and radio-therapeutic regimens show a significant enhancement in the activity compared with single treatment alone, either via a synergistic or additive effect. These strong biological activities observed in preclinical models, support the need for developing robust well-designed clinical studies, investigating the beneficial effects of the natural bioactive compounds alone or for combined therapies, on individual patients. These considerations suggest that much work remains to be done before RSV, EGCG and CUR can be considered as therapeutic agents for cancer therapy.

## Figures and Tables

**Table 1:** Cancer types and apoptotic pathways stimulated by RSV, EGCG, and CUR. ↑ (increase); ↓ (decrease).

Bioactive Compound	Cancer, Cell Types	Molecular Mechanisms	Apoptotic Pathways/Markers	Ref.
RSV	Chronic myeloid leukemia K562 cells	↑ pH2AX, ↑ p38MAPK, ↑ JNK, ↓ ERK, ↓ pAKT	Caspase-3 activation	[92]
RSV	Human glioma, U251 cells	↓ pAKT	Caspase-3 activation, LDH release	[93]
RSV	Prostate cancer, LNCaP cells	↑ FOXO, ↑ BIM, ↑ p27, ↓ PI3K/Akt/mTOR,↓ CD1	DR4, DR5 (extrinsic pathway)	[94]
RSV	Medulloblastoma, UW228 cells	↑ NF-Kβ ↑ Bcl-2	Annexin+	[95]
EGCG	Hepatocellular carcinoma cells	↑ BAX, ↑ p53, ↓ MMP ↓ Bcl-2, ↓ NF-Kβ	Caspase-3, -9 activation	[96]
EGCG	Human glioblastoma U87MG; T98G cells	↑ ROS, ↑ p38, ↑ JNKI, ↓ p-AKT, ↓ Bcl-2	Caspase-8 activation	[97]
EGCG	Bladder cancer, T24 cells	↓ PI3K/Akt, ↓ Bcl-2	Caspase-3 activation PARP cleavage	[98]
EGCG	Adult acute myeloid leukemia, AML cells	↑ ROS	Caspase-3, -8, -9 activation	[99]
EGCG	Bladder cancer, 5637, T4 cells	↑ BAX, autophagy related apoptosis	Caspase-3, -9 activation	[100]
EGCG	Colon cancer, (in vitro, in vivo models)	↓ Shh, ↓ PI3K	Caspase-3, -9 activation	[101]
EGCG	Colorectal cancer, HT29 cells	↑ ERS (Bip, pERK, eIF2α; IRIα)	Caspase-3, -7 activation	[102]
EGCG	Mesothelioma, REN and MM98 cell lines	↑GRP78, ↑ATF4, ERS induction	Caspase-3, -8 activation	[103]
CUR	Colon adenocarcinoma HCT-116 cells	↑ ROS	DR5 (extrinsic pathway)	[104]
CUR	Mouse myeloma cells	↑ p53, ↑ Hes family, ↑ BAX ↓ Bcl-2, ↓ NOTCH	Intrinsic pathway	[105]
CUR	Gastric cancer, SNU-1, SNU-5, and AGS cells	↓ Wnt/β-catenin	Annexin+	[106]
CUR	Hepatocellular carcinoma BCL-7402, QGY-7703 cells	↓ Wnt/β-catenin, ↓ VEGF, ↓ CD1	Annexin+	[107]
CUR	Thyroid cancer, BCPAP and TPC cells	↑ ROS, ↓ JAK/STAT	Caspase-3 activation	[108]
CUR	Melanoma vemurafenib-resistant cells (A375.S2)	↑ ROS, ↓ EGFR, ↓ AKT, ↓ MAPK	Caspase-3, -9 activation	[109]
CUR	Lung cancer, A-549 cells	↓ DJ-1, ↓ PI3K/Akt,	Annexin+	[110]

## Data Availability

Not applicable.

## Abbreviations

AKT	Protein-chinasi B
AMPK	Adenosine-monophosphate-activated protein kinase
AML	Acute promyelocytic leukemia
AP-1	Activating protein-1
APO-A1	Apolipoprotein A1
AR	Androgen receptor
ARH-I	Aplasia Ras homolog member I
ATF4	Activating transcription factor 4
BAD	BCL2-associated agonist of cell death
BAK	BCL-2 antagonist/killer
BAX	BCL-2-associated X protein
BCL-2	B-cell lymphoma-2
BCL-XL	B-cell lymphoma-extra large
BFL-1/A1	Bcl-2-related protein A1
BIM	BCL-2-interacting mediator of cell death
BIP	Immunoglobulin binding protein
BZM	Bortezomib
cAMP	Cyclic adenosine monophosphate
CD133	Prominin-1
CGE	Epigallocatechin
cGMP	Cyclic guanosine monophosphate
cIAP-2	Cellular inhibitor of apoptosis 2
COX	Cyclooxygenase
CSCs	Cancer stem cells
CUR	Curcumin
DR4	Death receptor 4
DR5	Death receptor 5
EGC	Epigallocatechin
EGCG	Epigallocatechin gallate
EGFR	Epidermal growth factor receptor
eIF2A	Eukaryotic translation initiation factor 2A
ER	Estrogen receptor
ERS	Endoplasmic reticulum stress
EZH2	Enhancer of zeste homolog 2
FADD	Fas-associated death domain
FAS	Fas cell surface death receptor
FASL	FAS ligand
FOXO1	Forkhead box protein O1
GA	Gallic acid
GRP78	Glucose-regulated protein, 78 kDa
HER2	Human epidermal growth factor receptor 2
IAPs	Inhibitors of apoptosis proteins
IC50	Half maximal inhibitory concentration
IGF-II	Insulin-like growth factor 2
IGFR	Insulin-like growth factor receptor
IR	Insulin receptor
IRE1a	Inositol-requiring enzyme-1a
JAK	Janus kinase
JAK	Janus kinase
JNK	c-Jun N-terminal kinase
LDH	Lattate dehydrogenase
LGR5	Leucine-rich repeat-containing G-protein-coupled receptor 5
LKB1	Live kinase B1
lncRNAs	Long non-coding RNAs
LPS	Lipopolysaccharide
MAPK	Mitogen-activated protein kinase
MAPKp38	p38 mitogen-activated protein kinases
MCL1	Myeloid cell leukemia-1
MMP	Mitochondrial membrane potential
mTOR	Mammalian target of rapamycin
NAC	N-acetylcysteine
ncRNAs	Non-coding RNAs
NF-BkB	Nuclear factor kappa B
p27/KIP1	Cyclin-dependent kinase inhibitor 1B
PARP-1	Poly [ADP-ribose] polymerase 1
PDCD4	Molecule of programmed cell death 4
PERK	Protein kinase RNA-like endoplasmic reticulum kinase
PI3K	Phoshatidylinositol-3 kinase
PIN1	Peptidyl-prolyl isomerase NIMA-interacting 1
PKA	Protein kinase A
PKB	Protein kinase B
PSA	Prostate specific antigen
PTEN	Phosphatase and tensin homolog
RASSF-1α	Ras association domain family 1 isoform A
ROS	Reactive oxygen species
RSV	Resveratrol
RTKs	Receptor tyrosine kinases
SHH	Sonic Hedgehog
SIRT1	Sirtuin 1
Sp1	Specificity protein 1
STAT1	Signal transducer and activator of transcription 1
STAT3	Signal transducer and activator of transcription 3
TNF	Tumor necrosis factor
TNF-α	Tumor necrosis factor α
TRAF2	TNF receptor-associated factor 2
VLDL	Very low-density lipoprotein
